# A Chromosome-Level Genome Assembly of *Toona ciliata* (Meliaceae)

**DOI:** 10.1093/gbe/evac121

**Published:** 2022-07-26

**Authors:** Xi Wang, Yu Xiao, Zi-Han He, Ling-Ling Li, Hui Yun Song, Jun-Jie Zhang, Xiang Cheng, Xiao-Yang Chen, Pei Li, Xin-Sheng Hu

**Affiliations:** College of Forestry and Landscape Architecture, South China Agricultural University, Guangzhou, 510642, China; Guangdong Key Laboratory for Innovative Development and Utilization of Forest Plant Germplasm, Guangzhou, 510642, China; College of Forestry and Landscape Architecture, South China Agricultural University, Guangzhou, 510642, China; Guangdong Key Laboratory for Innovative Development and Utilization of Forest Plant Germplasm, Guangzhou, 510642, China; College of Forestry and Landscape Architecture, South China Agricultural University, Guangzhou, 510642, China; Guangdong Key Laboratory for Innovative Development and Utilization of Forest Plant Germplasm, Guangzhou, 510642, China; College of Forestry and Landscape Architecture, South China Agricultural University, Guangzhou, 510642, China; Guangdong Key Laboratory for Innovative Development and Utilization of Forest Plant Germplasm, Guangzhou, 510642, China; College of Forestry and Landscape Architecture, South China Agricultural University, Guangzhou, 510642, China; Guangdong Key Laboratory for Innovative Development and Utilization of Forest Plant Germplasm, Guangzhou, 510642, China; College of Forestry and Landscape Architecture, South China Agricultural University, Guangzhou, 510642, China; Guangdong Key Laboratory for Innovative Development and Utilization of Forest Plant Germplasm, Guangzhou, 510642, China; College of Forestry and Landscape Architecture, South China Agricultural University, Guangzhou, 510642, China; Guangdong Key Laboratory for Innovative Development and Utilization of Forest Plant Germplasm, Guangzhou, 510642, China; College of Forestry and Landscape Architecture, South China Agricultural University, Guangzhou, 510642, China; Guangdong Key Laboratory for Innovative Development and Utilization of Forest Plant Germplasm, Guangzhou, 510642, China; College of Forestry and Landscape Architecture, South China Agricultural University, Guangzhou, 510642, China; Guangdong Key Laboratory for Innovative Development and Utilization of Forest Plant Germplasm, Guangzhou, 510642, China; College of Forestry and Landscape Architecture, South China Agricultural University, Guangzhou, 510642, China; Guangdong Key Laboratory for Innovative Development and Utilization of Forest Plant Germplasm, Guangzhou, 510642, China

**Keywords:** Meliaceae, *Toona ciliata*, Nanopore, Hi-C, phylogenetic evolution

## Abstract

*Toona ciliata* Roem is an important timber species in the *Toona* genus of the Meliaceae family and an endangered species due to over-cutting and a low rate of natural regeneration in China. Although molecular markers have been applied to studying population genetic diversity, the absence of a reliable reference genome limits in-depth genetic conservation and evolutionary studies of this species. Here, we reported a high-quality assembly of the whole genome sequence of *T. ciliata*. The total assembled genome has 520.64 Mb in length anchored on 28 chromosomes (contig N50 = 4.48 Mb). A total of 42,159 genes were predicted after the ab initio, homology-based, and transcriptome analyses. A total of 41,284 protein-encoding genes (97.92%) were functionally annotated and 1,246 non-coding RNAs were identified in the *T. ciliata* genome. Phylogenomic analysis showed that *T. ciliata* was divergent at 15.06 (6–25) Ma from *T. sinensis* of the same genus *Toona*. This whole genome sequence provides a valuable resource to study the genetic conservation and molecular evolution of *T. ciliata* in the future.

**Significance**

*Toona ciliata* is an important timber species in the genus *Toona* of the Meliaceae family and an endangered species at Grade II in China. Previous molecular and evolutionary studies of this species were restricted due to the absence of reference genome sequence. In this study, we sequenced the whole genome of *T. ciliata*, which would provide a valuable resource to study the evolution of *T. ciliata* and develop molecular markers for studying genetic conservation of this endangered species in the future.

## Introduction

*Toona ciliata* belongs to the monophyletic genus *Toona* of the Meliaceae family (Sun et al. 2014). The species, aka Chinese mahogany, is an important tropical and sub-tropical species and has great socio-economic values, such as the high-quality wood for furniture and the leaves for medicinal material (Edmonds 1993; Liao et al. 2009). It is naturally distributed in Pakistan and western India, Southeast Asia, southern China, Malaysia, and eastern Australia, and considered as an endangered species due to over-cutting and a low rate of natural regeneration (inbreeding depression) in China (Liang et al. 2011).

Previous studies on genetic diversity and molecular evolution of *T. ciliata* were based on molecular markers. Muellner et al. (2010) used the sequences of nuclear ITS and cpDNA segments (*trn*S-*trn*G, *psb*B, *psb*T, and *psb*N genes) to infer the evolutionary relationship of *T. ciliata* with other species of the Meliaceae family. Other molecular marker studies included the use of the sequence-related amplified polymorphisms and simple sequence repeats to analyze population genetic structure and mating systems. These studies indicated that *T. ciliata* had a high level of population genetic differentiation and significant effects of isolation by distance in its natural distribution in China (Li et al. 2015; Zhan et al. 2019), and a predominant outcrossing system, with selfing and inbreeding (Zhou et al. 2020). However, exploration of molecular markers is limited for our in-depth understanding of the molecular evolution of this species. Here, we reported the high-quality chromosome-level sequences of *T. ciliata* genome assembled by combining nanopore and Hi-C sequencing analyses. This is alternative to *T. sinensis* in the genus *Toona* whose genome sequence was recently assembled (Ji et al. 2021).

For a phylogenetic comparison, we selected four species used by Ji et al. (2021), including *Arabidopsis thaliana*, *Eucalyptus grandis*, *Salix purpurea*, and *Prunus persica*, and six different angiosperm plant species (*Citrus maxima*, *Citrus reticulata*, *Populus tremula*, *Glycine max*, *Amborella trichopoda*, and *T. sinensis*) for providing the further context of analysis. Although two species in the Meliaceae family, *Azadirachta indica* (Krishnan et al. 2012) and *Xylocarpus granatum* (GenBank accession: GCA_019650275.1), were sequenced, the downstream genomic analysis was limited because gene annotations (gff files) were not provided. These two species were not included for phylogenetic analysis. Our phylogenetic analysis helps to view the evolutionary divergence of *T. ciliata* from *T. sinensis* and other land plant species.

## Results and Discussion

### Genome Assembly

Genome survey was performed with *K*-mer analysis (*K* = 21) using three 350 bp-library datasets. The haploid genome size was estimated to be 253.36 Mb in length, and repetitive sequences accounted for 35.89% of the genome size. Genomic heterozygosity was estimated to be 11.90% and the GC content was 34.15%. The karyotype study confirmed that the sample tree has 56 chromosomes 2*n* = 56 (supplementary fig. S1, Supplementary Material online), consistent with the previous findings (Singh 1951; Styles and Vosa 1971; Mehra et al. 1972).

With the Nanopore sequencing platform (Biomarker Biotechnology Company, Beijing), we obtained 66.02 Gb raw sequence data. After filtering, we obtained 62.85 Gb clean data, with the sequencing depth of about 120.72×, the length of reads N50 of 26.95 kb, and the average read length of 20.25 kb. Distribution of the read sizes was summarized in supplementary table S1, Supplementary Material online.

After corrections of the clean data with Canu and the assembly contigs with Racon and Pilon, we obtained the genome of *T. ciliata*, which contained 324 contigs, with the N50 length of 4,484,018 bp and a total length of 520,643,266 bp. The GC content was 32.73% (Table 1).

**Table 1:** Summary of Genome Sequencing, Assembly, and Gene Annotations

Genome Assembly and Gene Annotations	Statistics
Genome assembly	
Number of contigs	349
Contig N50 (bp)	4,331,427
Contig N90 (bp)	600,000
Maximum contig size (bp)	12,652,477
Number of scaffolds	153
Scaffold N50 (bp)	17,615,381
Scaffold N90 (bp)	15,085,962
Maximum scaffold size (bp)	27,075,645
Genome size (bp)	520,643,266
Number of chromosomes	28
Total length of chromosomes (bp)	518,944,513
GC content (%)	32.73
Gene annotations	
Total number of genes	42,159
Number of GO annotation	34,439
Number of KEGG annotation	31,047
Number of KOG annotation	23,236
Number of Pfam annotation	34,769
Number of Swissprot annotation	33,407
Number of TrEMBL annotation	41,159
Number of eggNOG annotation	35,589
Number of NR annotation	41,216
Number of all protein-coding genes	41,284

**Fig. 1.**
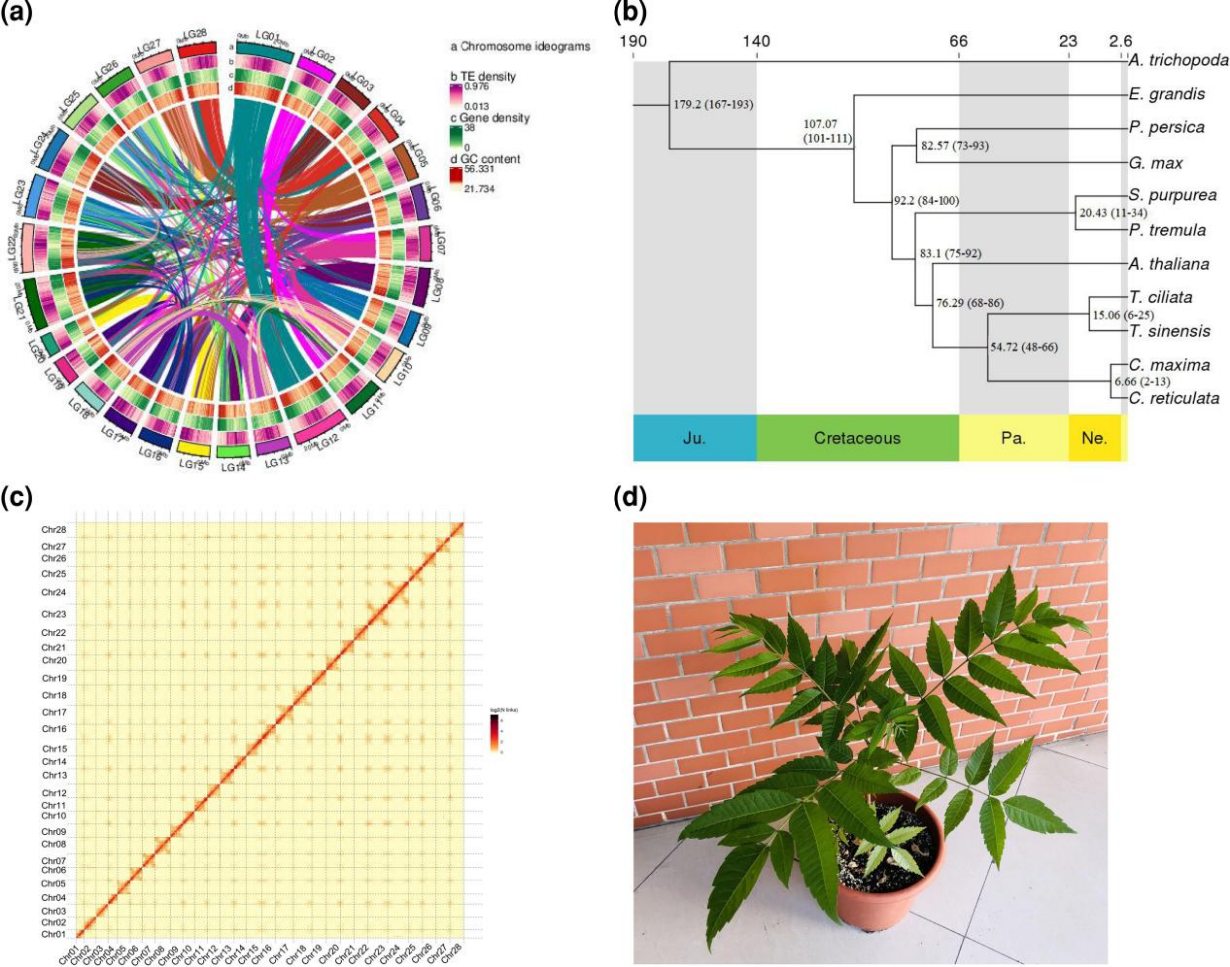
Chromosomal synteny, Hi-C heap map, phylogeny, and the sequenced plant clone of *Toona ciliata*. (*a*) A general view of *T. ciliata* genome and syntenic relationships within the genome. a, circular maps of 28 pseudochromosomes; b, the density distributions of TEs; c, distribution of gene density; d, GC distribution. (*b*) Heat map of the Hi-C interaction density among 28 pseudochromosomes. The chromosomal-level assembled genome of *T. ciliata* was segmented into 100-kb bins. The heatmap was used to visualize the number of interactions reported by Hi-C read pairs between each pair of bins. (*c*) Phylogenetic relationships among 11 species based on 1,276 single-copy genes. Species divergent times (95% CI) were estimated based on the phylogenetic relationships of 11 species, given the fossil records of 168–194 Ma of divergent time between *A. trichopoda* and *S. purpurea*, 100–111 Ma between *E. grandis* and *A. thaliana*, 51–85 Ma between *T. sinensis* and *C. maxima*, and 12–48 Ma between *P. tremula* and *S. purpurea*. These divergent times were derived from TIMETREE (http://www.timetree.org/) by loading the list of 11 species. (*d*) The sample clone of *T. ciliata* cultivated in a pot from which leaf samples were collected for genome sequencing.

Hi-C assembly with LACHESIS and manual adjustment and inspection were showed in supplementary table S2, Supplementary Material online. A total of 518,944,513 bp (99.67% of the total assembled genome) was anchored on 28 chromosome groups, with the scaffold N50 of 17,615,381 bp in length. Among the sequences located on chromosomes, the sequence length with the order and direction determined was 497,824,081 bp, accounting for 95.93% of the total length of the mapped chromosomes. Figure 1*a* shows the distribution of gene density, repeat sequence density, GC content, and collinearity within and among chromosomes of *T. ciliata*.

Figure 1 *b* shows the heat map analysis where 28 chromosomes were clearly distinguished. The completeness of genome assembly was assessed using CEGMA v2.5 and BUSCO v4.0 software with the eukaryotic core gene database and embryophyta_odb data set 9. A total of 450 CEGs were assembled with 98.25% completeness, and a total of 1,360 BUSCOs were assembled with 94.44% completeness, 1.11% fragmentation, and 4.44% missing.

### Gene Prediction and Annotations

Repeat annotation analysis showed a total of 253,150,116 bp of transposable elements (TEs) in the *T. ciliata* genome (supplementary table S3, Supplementary Material online), comprising 48.62% of the whole genome. Among all the classifications of TEs, long terminal repeats (LTRs) accounted for 42.13% of the whole genome and was the largest part of repeats. The length of tandem repeat sequences was 90,986,870 bp, accounting for 17.84%.

From the gene predictions of three approaches (ab initio, homology-based, and transcriptome), we obtained 42,159 coding genes, and most of them were derived from homologous and transcriptome prediction (supplementary table S4 and fig. S2, Supplementary Material online). The gene annotations were also evaluated by BUSCO analysis with embryophyta data set 9. Overall, 1,572 complete BUSCOs (97.40%) were identified in gene annotation, including 1,045 single-copy (64.75%), 527 duplicated BUSCOs (32.65%), and 22 fragmented BUSCOs (3.6%). In total, 20 genes (1.24%) were recognized as missing BUSCOs in our genome. The transcriptome data were evaluated by Hisat2, and 90.74% of RNA-seq clean data were mapped to our predicted exons.

A total of 41,284 protein-encoding genes (97.92%) were functionally annotated in the *T. ciliata* genome from alignment against public databases [NR, EggNOG, gene ontology (GO), Kyoto Encyclopedia of Genes and Genomes (KEGG), Swiss-Prot, Pfam, KOG, and TrEMBL] (Table 1). In addition, 1,246 non-coding RNAs were identified, including 676 tRNAs, 218 rRNAs, 149 miRNA, 72 snRNA and 131 snoRNA. Finally, a total of 148 pseudogenes were predicted, with 242,503 bp in total.

### Gene Family and Phylogenetic Analyses

A total of 37,030 gene families, containing 42,159 genes, were clustered in the *T. ciliata* genome together with the genomes of other 10 species (supplementary table S5, Supplementary Material online). A total of 4,517 gene families were common to all species. *Arabidopsis thaliana* had the most unique gene families (2,408), while *T. sinensis* had the least (442). *Toona ciliata* had 463 unique gene families (supplementary fig. S3, Supplementary Material online). Analysis of the copy number of gene families showed substantial differences among 11 species genomes (supplementary fig. S4, Supplementary Material online). *Toona ciliata* had more genes that had four or more copy numbers than *T. sinensis*.

A phylogenetic tree was constructed using 1,276 single-copy genes from the whole genome where divergent times among species were estimated using MCMCTree, calibrated with the known fossil records of three pairs of plant species divergent times (fig. 1*c*). The divergent time was 15.06 (6–25) Ma between *T. ciliata* and *T. sinensis*, and 76.29(75–92) Ma between *T. ciliata* and *A. thaliana*. Our estimates of the divergent times between *T. ciliata* and *T. sinensis* were overlapped with the previous results (7–49 Ma) derived from genetic markers (Muellner et al. 2010; Cavers et al. 2013; Koecke et al. 2013; Koenen et al. 2015). The divergent time was generally longer between *T. ciliata* and *T. sinensis* in genus *Toona* than between *C. maxima* and *C. reticulata* in genus *Citrus*, 6.66 (2–13) Ma. The divergent times between *T. sinensis* and *E. grandis* (101–111 Ma) were comparable with the results (107.7–111.9 Ma) of Ji et al. (2021). However, the divergent times between *T. sinensis* and *A. thaliana* were less than those obtained by Ji et al. (2021). As expected, *A. trichopoda*, the earliest divergent species in angiosperms, had the largest divergent times from all other species investigated. *Toona ciliata* was divergent at more than 80 Ma but <100 Ma from *P. tremula*, *S. purpurea*, *G. max*, and *P. persica*.

## Materials and Methods

### Sample Collection, DNA Extraction, and Genome Sequencing

The sample for genome sequencing was collected from one clone of the individual growing in Pupiao, Baoshan City, Yunnan Province, China (25.04N, 99.06E) and identified as *T. ciliata* var. *ciliata*. Figure 1*d* shows the individual cultivated in a pot in South China Agricultural University. Young and healthy leaves of the 1-year-old plant were collected for DNA isolation. Genomic DNA (gDNA) was extracted using the cetyltrimethylammonium bromide method (Doyle 1987). The concentration and purity of the gDNA were determined using a Nanodrop 2000 spectrophotometer and a Qubit fluorometer. DNA integrity was evaluated on a 0.5% agarose gel.

Genome sequencing and assembly were carried out by Biomarker Biotechnology Company in Beijing. The Nanopore reads were filtered and corrected using Canu (Koren et al. 2017). Nanopore sequencing library was constructed using a total of 9 μg gDNA to select larger fragment sizes (>10 kb) using a Blue Pippin Automatic Nucleic Acid Recovery System. The standard ONT library prep protocol was applied with a Ligation Sequencing Kit (SQK-LSK109) (Deamer et al. 2016). The raw reads were filtered with the thresholds of *Q*-value >7 and the minimum length of read fragments >500 bp. The high-quality reads were used to assemble the genome.

### Genome Size and Assembly

Preliminary genome survey was performed with *K*-mer analysis using three 350 bp-library datasets, including estimation of haploid genome size, proportion of repetitive sequences, genomic heterozygosity, and GC content. Our karyotype study was done to determine chromosomes of the sample.

We constructed Hi-C fragment libraries from 300 to 700 bp insert sizes (Vaser et al. 2017), and sequenced through Illumina platform. Adapter sequences of raw reads were trimmed, and low-quality pair-end reads were removed for clean data. The final valid reads were selected after the removal of the invalid read pairs, including dangling-end and self-cycle, re-ligation, and dumped products using Hic-Pro v2.10.0 (Servant et al. 2015).

The Hi-C data were mapped to these segments using BWA v0.7.10-r789 software. The uniquely mapped data were retained to perform assembly using LACHESIS (Burton et al. 2013) software. Parameters for running LACHESIS included: CLUSTER_MIN_RE_SITES = 100; CLUSTER_MAX_LINK_DENSITY = 2; ORDER_MIN_N_RES_IN_TRUNK = 110; ORDER_MIN_N_RES_IN_SHREDS = 104. After this step, placement and orientation errors exhibiting obvious discrete chromatin interaction patterns were manually adjusted.

The assembly results were assessed from three aspects: (1) the mapped rate (%) of clean reads on the reference genome sequence with bwa-mem software (Li 2013) for double-ended sequencing and bwa software for shorter sequences (Li and Durbin 2009); (2) the CEGMA(Core Eukaryotic Genes Mapping Approach) v2.5 (default parameters) database that contained 458 conserved key genes in eukaryotes (Parra et al. 2007) was used to evaluate the integrity of the final genome assembly; (3) BUSCOv4.0 software (Simão et al. 2015) was used to evaluate the integrity of the genome assembly by using OrthoDB V9 embryophyta database containing 1,440 conserved core genes. Parameters used with BUSCO were: –evalue 1e−03 (*E*-value cutoff for BLAST searches), -sp arabidopsis (reference species).

### Gene Annotations

We first customized a de novo repeat library of the genome using RepeatModeler, which can automatically execute two de novo repeat finding programs, including RECON v1.08 (Bao and Eddy 2002) and RepeatScout (Price et al. 2005). Then the full-length LTR retrotransposons (fl-LTR-RTs) were identified using both LTRharvest (Ellinghaus et al. 2008) (-minlenltr 100 -maxlenltr 40,000 -mintsd 4 -maxtsd 6 -motif TGCA -motifmis 1 -similar 85 -vic 10 -seed 20 -seqids yes) and LTR_finder (Xu and Wang 2007) (-D 40,000 -d 100 -L 9,000 -l 50 -p 20 -C -M 0.9). The high-quality intact fl-LTR-RTs and non-redundant LTR library were then produced by LTR_retriever (Ou and Jiang 2018). Non-redundant species-specific TE library was constructed by combining the de novo TE sequences library with the known Repbase v19.06 (Jurka et al. 2005), REXdb v3.0 (Neumann et al. 2019), and Dfam v3.2 (Wheeler et al. 2013) database. Final TE sequences were identified and classified by homology search against the library using RepeatMasker v4.10 (Tarailo-Graovac and Chen 2009). Tandem repeats were annotated by Tandem Repeats Finder (Benson 1999) and MIcroSAtellite identification tool (MISA v2.1) (Beier et al. 2017).

The tRNAscan-SE v1.3.1 (Lowe and Eddy 1997) was used to predict tRNA with eukaryote parameters. Identification of the rRNA genes was conducted by barrnap v0.9 (Loman 2017) with Rfam v12.0 (Griffiths-Jones et al. 2005). MiRNA was identified by searching miRBase (release 21) databases (Griffiths-Jones et al. 2006). The snoRNA and snRNA genes were predicted using INFERNAL (Nawrocki and Eddy 2013) against the Rfam (release 12.0) database.

Gene structure was predicted using three strategies: de novo, homologue-based, and transcriptomic analysis. The de novo gene models were predicted using two ab initio gene-prediction software tools, Augustus v2.4 (Stanke et al. 2008) and SNAP (Korf 2004). For the homolog-based approach, GeMoMa v1.7 (Keilwagen et al. 2016) software was performed by using reference gene model from the *T. sinensis*, *A. thaliana*, *Camellia sinensis*, *Acer yangbiense*, and *Pistacia vera*. For the transcript-based prediction, RNA-sequencing data were mapped to the reference genome using Hisat v2.0.4 (Kim et al. 2015) and assembled by Stringtie v1.2.3 (Pertea et al. 2015). GeneMarkS-T v5.1 (Tang et al. 2015) was used to predict genes based on the assembled transcripts. The PASA v2.0.2 (Haas et al. 2003) software was used to predict genes based on the unigenes [and full-length transcripts from the PacBio (ONT) sequencing] assembled by Trinity v2.11. Gene models from these different approaches were combined using the EVM v1.1.1 (Haas et al. 2008) and updated by PASA. The gene annotations were obtained with BLASTv2.2.31 (*E*-value = 1*e*−5) by aligning against the GenBank Non-Redundant (NR, 20200921), TrEMBL (202005), Pfam (33.1), SwissProt (202005), eukaryotic orthologous groups (KOG, 20110125), (GO, 20200615), KEGG (20191220) databases.

GenBlastA v1.0.4 program (She et al. 2009) was used to scan the whole genome after masking the predicted functional genes. Putative candidates were then analyzed by searching for non-mature mutations and frame-shift mutations using GeneWise v2.4.1 (Birney et al. 2004).

### Gene Family and Phylogenetic Analyses

We compared genomic sequences of 11 species (*A. thaliana*, *A. trichopoda*, *C. maxima*, *C. reticulata*, *E. grandis*, *G. max*, *P. persica*, *P. tremula*, *S. purpurea*, *T. sinensis*, and *T. ciliata*), with an emphasis on the evolutionary divergence between *T. ciliata* and *T. sinensis*. Genome sequences of these species except *T. ciliata* were downloaded from different databases (supplementary table S6, Supplementary Material online).

The protein sequences of 11 species were classified by gene family with Orthofinder V2.4 software (Emms and Kelly 2019), and the comparison method was diamond while *E*-value was 0.001. PANTHER V15 database (Mi et al. 2019) was used to annotate the obtained gene families. GO and KEGG enrichment analyses were carried out for the gene family unique to *T. ciliata* by clusterProfile v3.14.0 (Yu et al. 2012).

The predicted protein sets were condensed to include a single peptide sequence for each gene by filtering out redundant alternative splicing events with Gblocks V0.91 (Talavera and Castresana 2007). The single-copy genes were used to construct phylogenetic tree by the ModelFinder (Kalyaanamoorthy et al. 2017), and the optimal model was JTT + F+I + G4 with the maximum likelihood (ML) method. The number of bootstraps was set to 1,000. By combining the known divergent times of multiple species derived from TIMETREE (http://www.timetree.org/), the divergent times among species were calculated using the MCMCTREE module in PAML v4.9 (Yang 1997; Puttick 2019).

## Supplementary Material

evac121_Supplementary_Data

## Data Availability

The data reported in this study are available under accession no. CNP0001985 in the CNGB Nucleotide Sequence Archive (CNSA: https://db.cngb.org/search/project/CNP0001985/), including raw sequence reads, genome assembly files, gene annotations, pseudogene predictions, and ncRNA files.
