# Supplementary material for: A Chromosome-Level Genome Assembly of Toona ciliata (Meliaceae)
Source: Genome Biol Evol. 2022 Jul 26;14(8):evac121. doi: 10.1093/gbe/evac121 (PMC9348625; doi:10.1093/gbe/evac121)
Supplement: evac121_Supplementary_Data [file evac121_supplementary_data.docx]

**Supplementary Material**

**A Chromosome-Level Genome Assembly of *Toona ciliata* (Meliaceae)**

Xi Wang^1,2^, Yu Xiao^1,2^, Zi-Han He^1,2^, Ling-ling Li^1,2^, Hui-Yun Song ^1,2^, Jun-Jie Zhang^1,2^, Xiang Cheng ^1,2^, Xiao-Yang Chen^1,2^, Pei Li^1,2*^, Xin-Sheng Hu^1,2*^

1. College of Forestry and Landscape Architecture, South China Agricultural University, Guangdong, China

2. Guangdong Key Laboratory for Innovative Development and Utilization of Forest Plant Germplasm, Guangdong, 510642 Guangdong, China

* Correspondence: Pei Li, [lipei-meinv@163.com](mailto:lipei-meinv@136.com);

Xin-sheng Hu，[xinsheng@scau.edu.cn](mailto:xinsheng@scau.edu.cn)

**This file includes:**

Figures S1 to S4

Tables S1 to S7

**
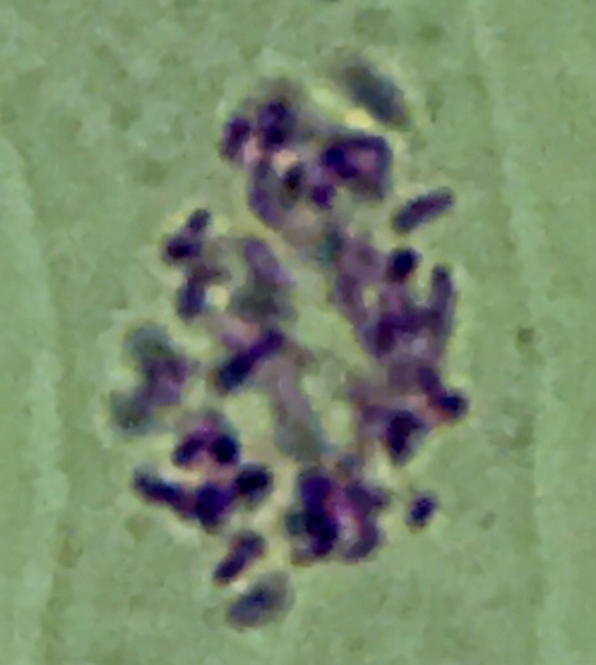
****
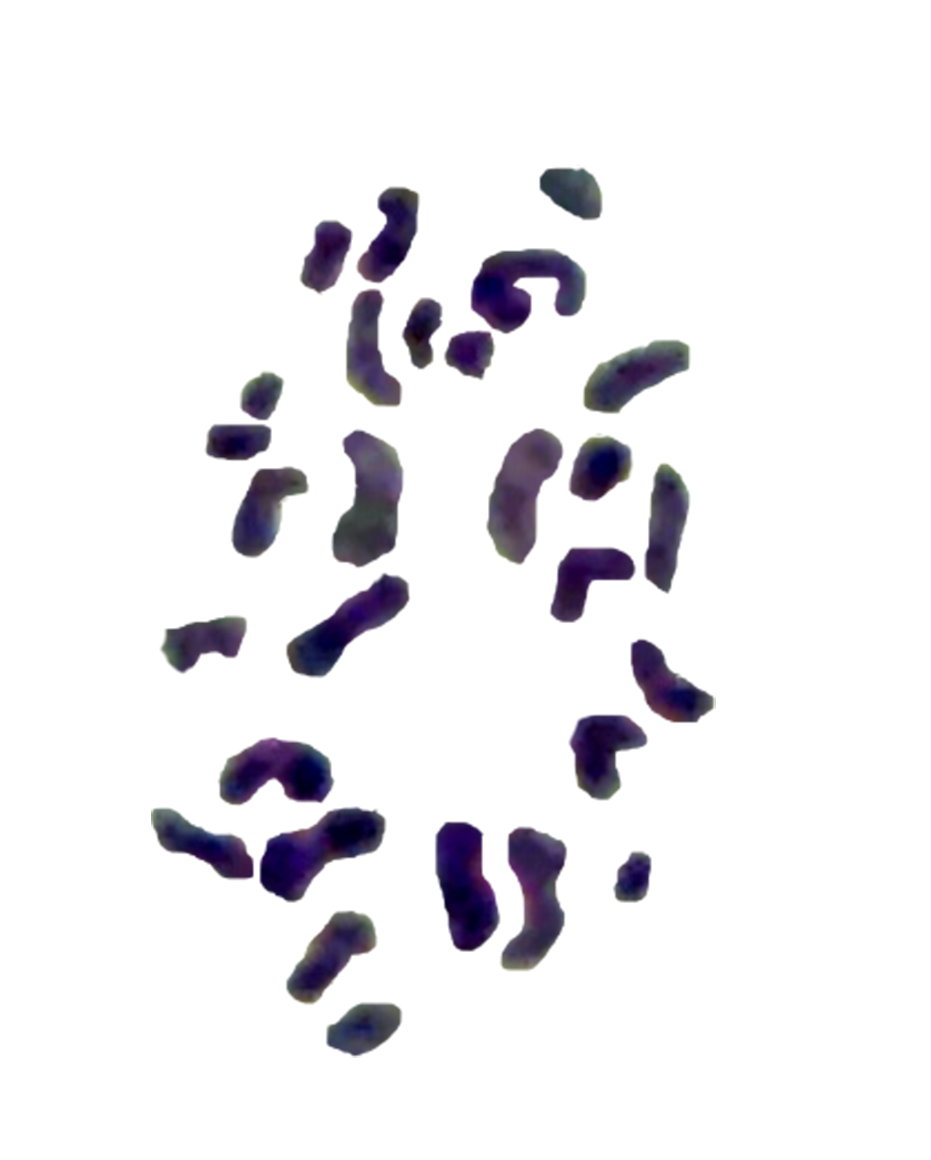
**

**Supplementary Figure S1.** **A micrograph (100×) for the karyotype examination of the sample tree**. The micrograph shows that twenty-eight pairs of chromosomes were observed in a cell of *T. ciliata*.

**
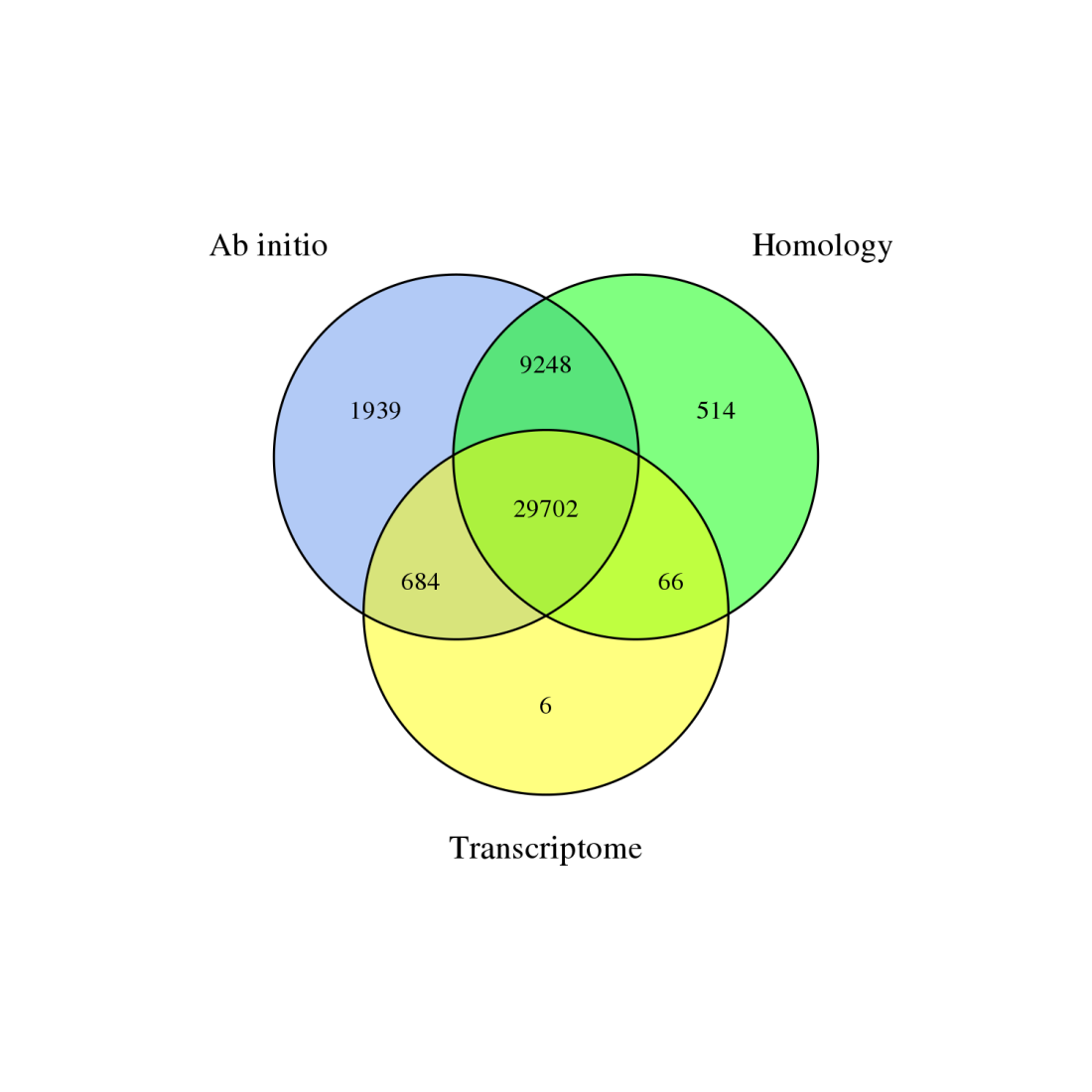
**

**Supplementary Figure S2. A map for the number of genes in *Toona ciliata* genome predicted by three approaches.** Three approaches of gene predictions were in different colors: ab initio, homology-based and transcriptome. The values in overlapping areas were the numbers of genes commonly predicted by different approaches.


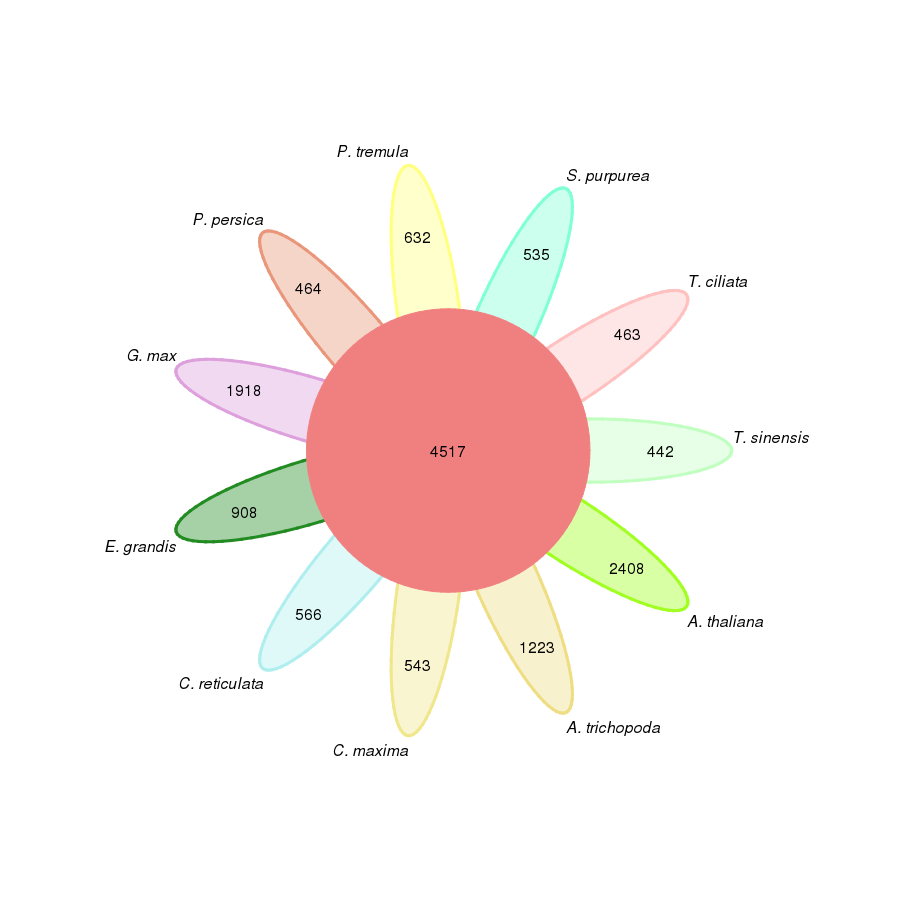


**Supplementary Figure S3. Cluster analysis of gene families of *T. ciliata* and other 10 species.** The numbers of gene families in the center circle were common to all species, and the numbers of gene families on each edge were specific to each species.


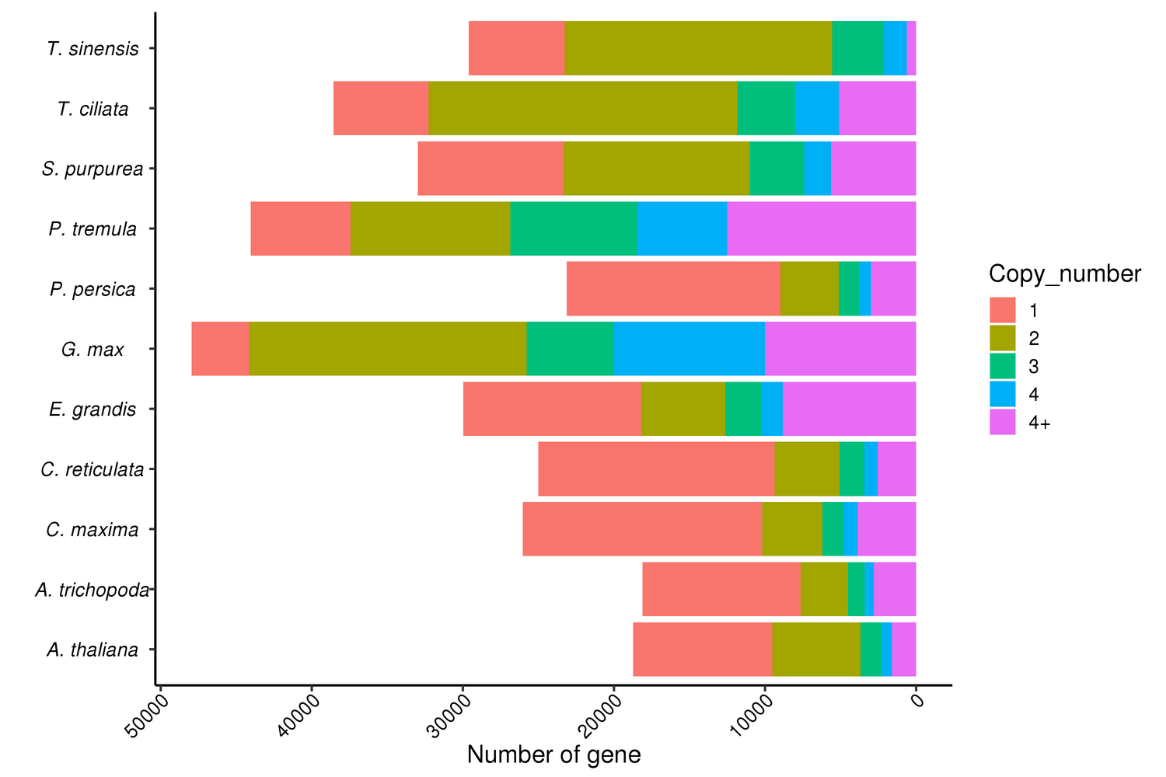


**Supplementary Figure S4.** **Comparison of gene copy numbers among 11 species.** The numbers of gene copies ranging from 1 to 4-plus were present in different colors in the bar chart.

**Supplementary Table S1.** **Summary of the read length distribution of *T. ciliata***

| **Length** | **Reads number** | **Total length** | **Percentage** | **Average length** |
| --- | --- | --- | --- | --- |
| 2000~5000 | 307,596 | 1,042,593,011 | 1.65% | 3,389.48 |
| 5000~10000 | 443,286 | 3,499,569,594 | 5.56% | 7,894.60 |
| 10000~20000 | 1,118,639 | 16,191,022,610 | 25.76% | 14,473.85 |
| 20000~30000 | 606,413 | 14,841,629,052 | 23.61% | 24,474.45 |
| 30000~40000 | 316,945 | 10,921,903,972 | 17.37% | 34,459.93 |
| 40000~50000 | 164,731 | 7,322,763,702 | 11.65% | 44,452.85 |
| 50000~60000 | 83,101 | 4,518,824,263 | 7.18% | 54,377.49 |
| 60000~70000 | 37,425 | 2,405,812,652 | 3.82% | 64,283.57 |
| 70000~80000 | 15,410 | 1,143,935,367 | 1.82% | 74,233.31 |
| >=80000 | 10,392 | 962,150,210 | 1.53% | 92,585.66 |

**Supplementary Table S2. Twenty-eight chromosomes derived from Hi-C assembly of *T. ciliata*** **genome**

| **Group** | **Cluster number** | **Cluster length (bp)** | **Order number** | **Order length (bp)** |
| --- | --- | --- | --- | --- |
| Chr01 | 14 | 11,199,069 | 8 | 10,039,615 |
| Chr02 | 20 | 16,234,215 | 15 | 15,444,654 |
| Chr03 | 8 | 15,888,025 | 5 | 15,385,864 |
| Chr04 | 5 | 12,050,165 | 5 | 12,050,165 |
| Chr05 | 18 | 17,925,651 | 11 | 16,325,559 |
| Chr06 | 15 | 16,178,214 | 11 | 15,633,244 |
| Chr07 | 9 | 17,039,488 | 5 | 16,103,085 |
| Chr08 | 23 | 21,986,271 | 13 | 19,976,366 |
| Chr09 | 13 | 17,043,998 | 9 | 16,135,027 |
| Chr10 | 7 | 15,472,040 | 4 | 15,085,662 |
| Chr11 | 11 | 17,445,779 | 4 | 16,299,248 |
| Chr12 | 17 | 17,034,855 | 11 | 16,399,109 |
| Chr13 | 9 | 18,265,586 | 7 | 18,063,620 |
| Chr14 | 10 | 16,201,138 | 6 | 15,577,693 |
| Chr15 | 17 | 20,994,639 | 13 | 20,209,528 |
| Chr16 | 11 | 18,575,894 | 6 | 17,614,881 |
| Chr17 | 11 | 22,862,221 | 10 | 22,656,303 |
| Chr18 | 16 | 24,637,156 | 12 | 24,052,182 |
| Chr19 | 9 | 18,564,181 | 6 | 18,224,937 |
| Chr20 | 11 | 19,563,137 | 6 | 18,405,166 |
| Chr21 | 13 | 17,785,524 | 9 | 17,200,035 |
| Chr22 | 13 | 18,960,934 | 8 | 17,905,720 |
| Chr23 | 8 | 26,270,511 | 3 | 25,471,093 |
| Chr24 | 8 | 27,734,559 | 5 | 27,075,245 |
| Chr25 | 13 | 18,554,336 | 8 | 17,645,789 |
| Chr26 | 11 | 18,245,493 | 7 | 17,235,641 |
| Chr27 | 13 | 18,676,157 | 10 | 18,053,373 |
| Chr28 | 7 | 17,555,277 | 7 | 17,555,277 |
| Total (Ratio %) | 340 (97.42) | 518,944,513 (99.67) | 224 (65.88) | 497,824,081 (95.93) |

**Supplementary Table S3. Classification of annotated repeat sequences in *T. ciliata* genome**

| **Type** | **Number** | **Length(bp)** | **Rate(%)** |
| --- | --- | --- | --- |
| DNA | 101,818 | 27,248,746 | 5.23 |
| DIRS | 771 | 206,359 | 0.04 |
| LINE | 20,404 | 5,923,236 | 1.14 |
| SINE | 2,385 | 395,808 | 0.08 |
| LTR | 273,919 | 219,375,967 | 42.13 |
| Total | 399,297 | 253,150,116 | 48.62 |

**Supplementary Table S4.** **Summary of the gene predictions using three approaches**

| **Method** | **Software** | **Species** | **Gene number** |
| --- | --- | --- | --- |
| Ab initio | Augustus | - | 34,565 |
|  | SNAP | - | 54,795 |
| Homology-based | GeMoMa | *A. thaliana* | 33,750 |
|  |  | *A. yangbiense* | 36,293 |
|  |  | *C. sinensis* | 38,505 |
|  |  | *P. vera* | 39,527 |
| RNAseq | GeneMarkS-T | - | 24,870 |
|  | PASA | - | 27,707 |
| Integration | EVM | - | 42,159 |

**Supplementary Table S5.** **Cluster analysis of gene families in *T. ciliata* and other 10 species***

| **Item** | **Ath** | **Atr** | **Cma** | **Cre** | **Egr** | **Gma** | **Pep** | **Ptr** | **Spu** | **Tci** | **Tsi** |
| --- | --- | --- | --- | --- | --- | --- | --- | --- | --- | --- | --- |
| Number of genes | 27,624 | 26,846 | 30,119 | 28,606 | 36,348 | 56,044 | 26,873 | 47,601 | 35,125 | 42,159 | 34,345 |
| Number of genes in orthogroups | 18,736 | 18,114 | 26,025 | 25,001 | 29,975 | 47,957 | 23,122 | 44,039 | 32,981 | 38,559 | 29,605 |
| Number of unassigned genes | 8,888 | 8,732 | 4,094 | 3,605 | 6,373 | 8,087 | 3,751 | 3,562 | 2,144 | 3,600 | 4,740 |
| Percentage of genes in orthogroups | 67.8 | 67.5 | 86.4 | 87.4 | 82.5 | 85.6 | 86 | 92.5 | 93.9 | 91.5 | 86.2 |
| Percentage of unassigned genes | 32.2 | 32.5 | 13.6 | 12.6 | 17.5 | 14.4 | 14 | 7.5 | 6.1 | 8.5 | 13.8 |
| Number of orthogroups containing species | 12,968 | 12,836 | 18,891 | 18,859 | 16,585 | 18,657 | 17,067 | 18,078 | 18,017 | 19,076 | 16,827 |
| Percentage of orthogroups containing species | 35 | 34.7 | 51 | 50.9 | 44.8 | 50.4 | 46.1 | 48.8 | 48.7 | 51.5 | 45.4 |
| Number of species-specific orthogroups | 2,408 | 1,223 | 543 | 566 | 908 | 1,918 | 464 | 632 | 535 | 463 | 442 |
| Number of genes in species-specific orthogroups | 6,567 | 4,798 | 1,750 | 1,594 | 4,503 | 7,563 | 1,856 | 1,836 | 2,885 | 2,307 | 961 |
| Percentage of genes in species-specific orthogroups | 23.8 | 17.9 | 5.8 | 5.6 | 12.4 | 13.5 | 6.9 | 3.9 | 8.2 | 5.5 | 2.8 |

* Ath: *Arabidopsis thaliana*; Atr: *Amborella trichopoda*; Cma: *Citrus maxima*; Cre: *Citrus reticulata*; Egr: *Eucalyptus grandis*; Gma: *Glycine max*; Pep: *Prunus persica*; Ptr: *Populus tremula*; Spu: *Salix purpurea*; Tci: *Toona ciliata*; Tsi: *Toona sinensis*.

**Supplementary Table S6. Websites for downloading genome sequences of 10 plant species used in this study**

| Species | Download |
| --- | --- |
| *Citrus maxima* | http://citrus.hzau.edu.cn/orange/download/HWB.chromosome.fa.tar.gz |
| *Populus tremula* | http://128.192.158.63/index.php/databases/spta-717-genome |
| *Salix purpurea* | https://phytozome-next.jgi.doe.gov/info/Spurpurea_v5_1 |
| *Eucalyptus grandis* | https://phytozome.jgi.doe.gov/pz/portal.html#!info?alias=Org_Egrandis |
| *Amborella trichopoda* | https://phytozome.jgi.doe.gov/pz/portal.html |
| *Prunnus persica* | http://genome.jgi.doe.gov/pages/dynamicOrganismDownload.jsf?organism=Ppersica |
| *Citrus reticulata* | http://citrus.hzau.edu.cn/orange/download/index.php |
| *Arabidopsis thaliana* | ftp://ftp.ensemblgenomes.org/pub/plants/release-45/fasta/arabidopsis_thaliana |
| *Glycine max* | https://phytozome.jgi.doe.gov/pz/portal.html#!info?alias=Org_Gmax |
| *Toona sinensis* | https://db.cngb.org/search/project/CNP0000958/ |
